# Conflict, violence, and warfare among early farmers in Northwestern Europe

**DOI:** 10.1073/pnas.2209481119

**Published:** 2023-01-17

**Authors:** Linda Fibiger, Torbjörn Ahlström, Christian Meyer, Martin Smith

**Affiliations:** ^a^School of History, Classics and Archaeology, University of Edinburgh, Edinburgh EH8 9AG, Scotland; ^b^Department of Archaeology and Ancient History, Lund University, Lund SE-221 00, Sweden; ^c^OsteoARC - OsteoArchaeological Research Centre, Goslar 38644, Germany; ^d^Department of Archaeology and Anthropology, Forensic and Biological Anthropology, Bournemouth University, Poole BH12 5DD, United Kingdom

**Keywords:** Neolithic Europe, violence and conflict, warfare, bioarchaeology

## Abstract

Bioarchaeological evidence of interpersonal violence and early warfare presents important insights into conflict in past societies. This evidence is critical for understanding the motivations for violence and its effects on opposing and competing individuals and groups across time and space. Selecting the Neolithic of northwestern Europe as an area for study, the present paper examines the variation and societal context for the violence recorded in the human skeletal remains from this region as one of the most important elements of human welfare. Compiling data from various sources, it becomes apparent that violence was endemic in Neolithic Europe, sometimes reaching levels of intergroup hostilities that ended in the utter destruction of entire communities. While the precise comparative quantification of healed and unhealed trauma remains a fundamental problem, patterns emerge that see conflict likely fostered by increasing competition between settled and growing communities, e.g., for access to arable land for food production. The further development of contextual information is paramount in order to address hypotheses on the motivations, origins, and evolution of violence as based on the study of human remains, the most direct indicator for actual small- and large-scale violence.

## Significance

This paper explores the key role bioarchaeology plays in creating meaningful perspectives on human conflict and the emergence of warfare in Neolithic Europe. Skeletal datasets are considered in the context of social, economic, and demographic changes that accompanied the shift to a sedentary farming economy. Increasing competition and inequality are key factors that fostered the emergence of larger-scale human conflict and warfare. Beyond numbers, these insights should allow for more significant engagement with the unique experiential qualities of violence in prehistory.

Discussions on interpersonal violence and conflict in Neolithic Europe have shifted from past assumptions on the generally peaceful nature of the period (e.g., refs. (1) and (2)) to a recognition of physical violence as a widespread feature that was not limited to one-off violent events (3–5). The Neolithic is defined here as the period between the mobile or semi-mobile hunter-fisher-gatherer groups of the Mesolithic, and the frequent appearance of metal objects (including purpose-made metal weaponry). The advent of individuals ascribed the social status of specialist warriors as a recognizable social and material cultural concept signals the start of the Bronze Age (6, 7). The European Neolithic sees the emergence of the farming lifestyle, domestication, sedentism, monumentality, and a significant population increase (8). These changes provide the context and most probably some of the impetus for transformations in the nature and scale of violent interaction during the period, involving scale, tactics, objectives, and inferred social significance.

**Fig. 1.**
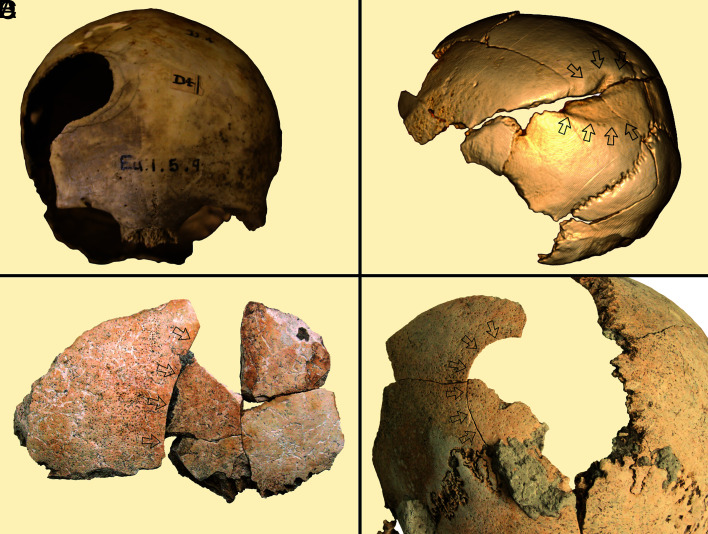
Examples of blunt force trauma in Neolithic crania from Northwestern Europe: (*A*) Belas Knap, England (unhealed); (*B*) Schöneck-Kilianstädten, Germany (healed); (*C* and *D*) Halberstadt, Germany (unhealed).

**Fig. 2.**
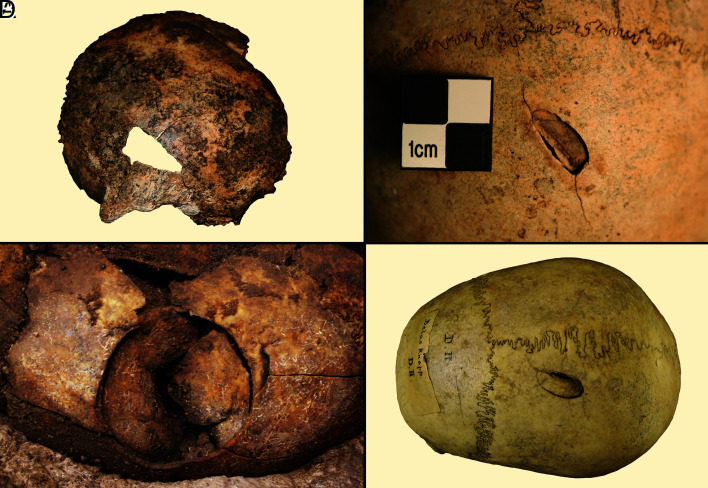
Examples of unhealed penetrating sharp/blunt force injuries caused by stone axes or club-like implements: (*A*) Bredelem, Germany; (*B*) Raevehoj, Denmark; (*C*) Salzmünde, Germany; (*D*) Belas Knap, England.

**Fig. 3.**
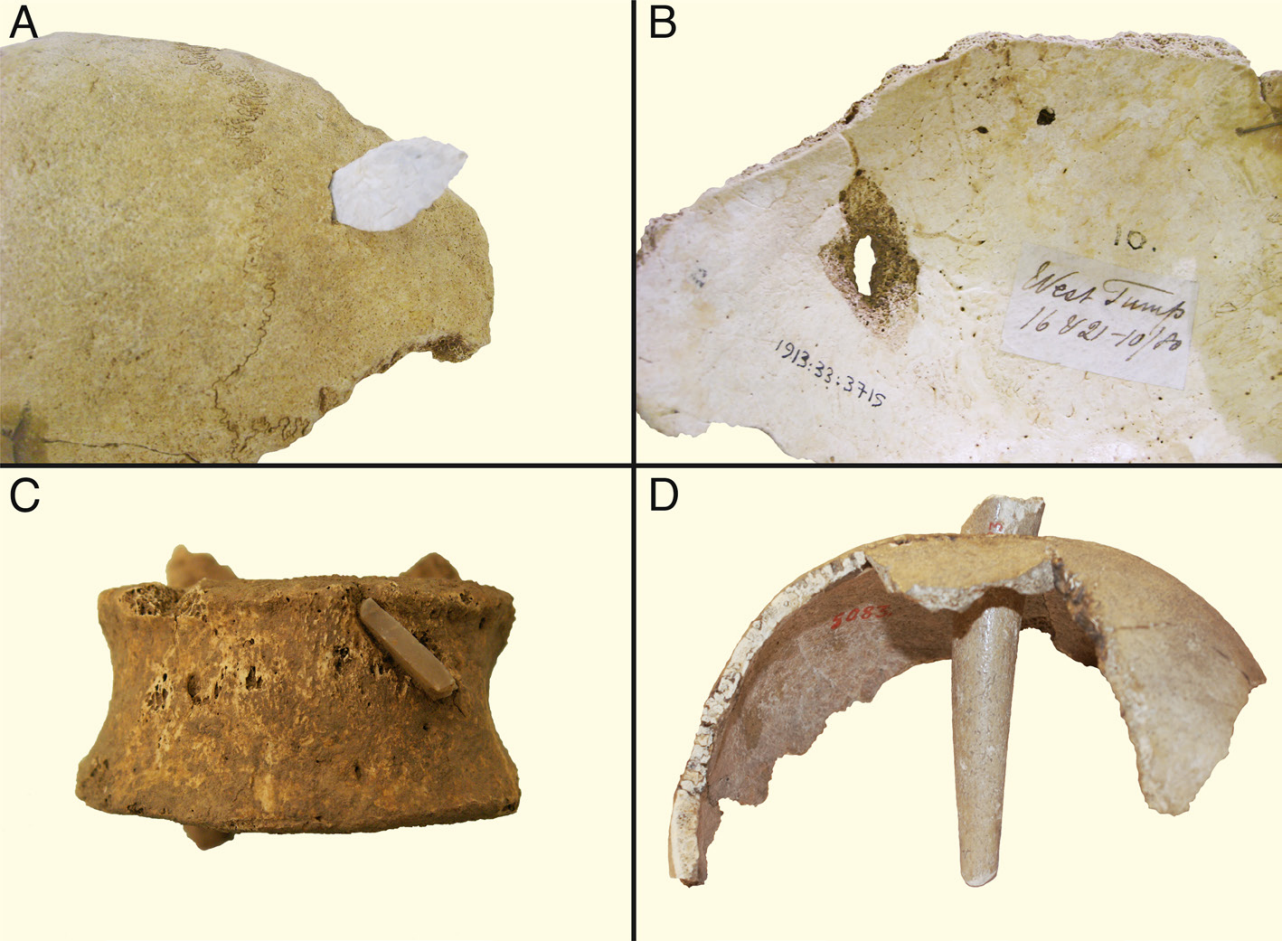
Examples of unhealed penetrating injuries with embedded points: (*A*) leaf-shaped arrowhead refitted to elliptical penetrating defect in a cranium from West Tump, England; (*B*) endocranial view of the same defect showing internal beveling; (*C*) arrowhead embedded in a vertebra from Eulau, Germany; (*D*) antler tine embedded in a cranium from Tygelsjö, Sweden.

Technology certainly is another unifying factor across a region where the chronological framework for the Neolithic diverges widely, with 7th millennium BC dates for the earliest Neolithic in Southeast Europe contrasting with the much later 4th millennium Neolithic dates for Scandinavia (9–10). The weapon tool technology of the European Neolithic includes stone axes, adzes and arrowheads, flint knives, wooden and stone-headed clubs, as well as antler picks and sling shot. Some implements, such as stone adzes, may also be symbols of identity and social differentiation (11). The available weaponry determines the nature of skeletal evidence for violence recognized for the period, which is often dominated by blunt force trauma, but also sees the occurrence of more penetrative injuries caused by projectiles and the widely prevalent stone axes and adzes (Figs. 1–3). With their combination of a sharp cutting edge and considerable weight and mass, defects resulting from adzes and axes can have both blunt and sharp force characteristics, and often leave distinctive fracture patterns (12, 13), while projectile injuries may be highly visible when arrowheads remain embedded in the bone (14, 15). In contrast, blunt force trauma presents particular challenges when trying to identify what implement may have caused a given injury, though more recently experimental bioarchaeology has been used to establish fracture patterns for particular weapon tools (e.g., refs. (16) and (17)).

Such (probable) weapons or the form and arrangement of built structures make undoubted contributions to debates regarding the existence of prehistoric violence, but these lines of evidence inevitably produce equivocal conclusions. Suggestions that the intended purpose of flaked stone points or earthwork enclosures might relate to conflict are innately open to counter interpretation, to the extent that it is possible to project on to the evidence whatever kind of Neolithic an author happens to favor. Human remains are ultimately far less equivocal in offering opportunities to quantify patterns in the data at an aggregate level across wide regions. Depressed cranial fractures, above the hat brim line (i.e., above the line of an imaginary hat, identifying a location on the superior half of the cranium) (18)), defense (parry) fractures of the distal ulna, and even stone points embedded in the bone can be explained as resulting from accidents on occasion, although the current authors are unaware of any published clinical studies that would lend support to such interpretations. When occurring in the numbers and proportions cited in the current article, the most plausible explanation for the injury patterns seen is intentional violence perpetrated between groups and individuals.

Given the nature of the archaeological skeletal record (i.e., it is never complete) and the fact that skeletal signs of injury only ever reflect the minimum number of injuries present originally, the endemic levels of violence suggested for some regions of Europe are a real feature of this period, rather than based on a sampling bias. Overall, in most Neolithic societies from Europe, a crude prevalence of up to ca. 10% or more of individuals with evidence of trauma seems to be quite common (Table 1). Men are more frequently affected than women or children (e.g., (19), (20)), but these figures tend to include all injuries, from targeted lethal axe or adze strikes to the head to small depressions possibly caused by everyday mishaps. In contrast, trauma prevalence rates of around 50% are recorded from the victims of mass violence sites (21, 22).

**Table 1.** Regional skeletal trauma data for the European Neolithic

	% of individuals with cranial trauma				
Region	Nonlethal	Lethal	Total	N	Source
Britain	7.2	5	12.2	456	Wysocki and Schulting (2005) (23); Smith (2013) (4); Schulting and Fibiger (2014) (24); Smith (2017) (25)
Denmark	12.6	4.6	16.9	261	Fibiger et al*.* (2013) (3)
France	4.2	3.2	7.4	687	Schulting n.d.
Germany	4.4	3.5	7.6	634	Fibiger (2018) (26)
N.Spain	11.5	0.5	12	208	Vegas et al*.* (2012) (15)
Sweden	6.8	2.6	9.4	117	Fibiger et al*.* (2013) (3)
Mean	7.78	3.23	10.91		
Total				2,363	

The extent to which these figures represent an increase on preceding periods is challenging to quantify, principally due to human burials being even more scarce from periods predating domestication and often subject to worse preservation than those of Neolithic groups. The most comprehensive study to date of violence among European hunter-gatherers by Estabrook (27) explores the challenges of assessing whether injuries to bone were the result of deliberate violence. She lists 77 Mesolithic instances of bony injury attributed to violent assaults, of which 46 affect the skull/cranium. These numbers are difficult to compare to those presented in the current study as they are not framed in proportional/percentage terms of the wider sample they are drawn from. In commenting on Estabrook’s figures, Waller (28) estimates the total sample as approximating 2,000 individuals. This number would give a crude percentage rate of 3.85% overall and 2.3% for cranial injuries. This latter figure is not directly comparable to that of the current study as it does not reference how many of the 2,000 burials had surviving crania, with the majority acknowledged to consist of “only fragments of bone…often damaged by taphonomic processes” (28). According to the best evidence currently available, there would therefore appear to have been a genuine increase in the prevalence of violence following domestication, notwithstanding issues of preservation.

This raises the question whether it is possible to start using the term war for some of the violent interactions of the period––and indeed recognize it (bio-)archaeologically when characterizing the skeletal evidence for violence that survives. Increasing territoriality related to crop farming and pastoralism and varied and seasonal surplus production and inevitable increases and variation in group size, organization, and economic inequality are probably all contributing factors (5). At the same time, it is important to remember that violence, as a form of communication, is not a new feature of the period. While endemic levels of violence are certainly present within the period and region, this does not equate to experiential or numerical uniformity, nor does it allow for the characterization of the whole period as particularly belligerent in the context of prehistoric Europe, or even human history as a whole (29). What we do see is an increase in significant violent events resulting in mass fatalities, which cluster in the earliest Neolithic of Central Europe in particular (30). This evidence for sustained, larger-scale intergroup violence may indeed signal the more permanent inauguration of warfare in social relations in the face of unprecedented and complex societal, economic, and demographic changes.

## Identifying Violence and Conflict in Neolithic Europe: Complex Skeletal Datasets

The scale and impact of conflict are most reliably investigated through the bioarchaeological record. Skeletal signs of violent injury, considered at a local and regional level, present the best and most immediate evidence of violence in prehistory (31). This is important as unambiguous weapons of violence are largely absent across Neolithic Europe, whereas secondary indicators of potential conflict, such as defensive sites or enclosures, are not ubiquitous (3, 32). Nonnormative or unusual mortuary treatment may also signpost the presence of a violent event, bearing in mind that not all injuries leave traces on the skeleton. A frequently cited study by Milner (33) on 19th-century arrow injuries, for example, states that only one-third of such wounds hit a bone. For the Neolithic context, this would mean that approximately 70% of arrow injuries and 50% of overall bodily trauma remain invisible in skeletal remains (30). This is a stark reminder that even when there is no skeletal evidence for violence, it may still have occurred and was likely more frequent than is visible to us today. This is also emphasized by the fact that even at clearly identified sites of mass violence, such as the Linearbandkeramik (LBK) site of Talheim (Germany), physical signs of violence were not present on all individuals in the mass grave (22).

Identifying not only intent but also intended lethality can be challenging. Many (though by no means all) violence-related population and regional studies for the period focus on the cranium as a well-established focus for intentional injuries (34) and separately consider healed versus unhealed trauma. While healing does not confirm the intention to harm rather than kill, together with a detailed assessment of injury morphology, size, and location, it may offer potential insights into tactics, attacker/victim constellation, and consequences of an injury for the individual. Further exploration of age and potentially gendered patterns may also suggest that a section or sections of the population were specifically targeted, or reveal differences in the ability and competence of individuals to engage in violent behavior. This, in turn, offers insights into potential division of labor as related to violence as social interaction and comparisons across space and time (3, 35).

As the Neolithic covers a timespan of several thousand years, there inevitably is variation, with episodes and regions of increased violence, and others which show less or no obvious skeletal evidence for violent interaction. The challenge is gathering enough reliable baseline data (i.e., true prevalence) for valid comparisons of the level of physical violence experienced. Unfortunately, most older examinations of Neolithic skeletal remains can be unreliable in terms of the assessment of healed or unhealed injuries, if they report population data at all. In contrast, more recent studies consistently identify new lesions while at the same time rectifying older misinterpretations (e.g., ref. (23)). To gain a good understanding of the level of violence-related injuries present in a population, it is imperative to look at all site types with human skeletal remains, which many of the more recent studies have done (e.g., refs. (3, 4, and 25)). Consideration of violent injury from across the mortuary record has the potential to yield important insights into the day-to-day level of violence present and prevents bias toward the more spectacular mass fatality sites. Unfortunately, the problem of comparability is further confounded by differing funerary customs.

The mortuary record for the period is to some extent fragmented and incomplete due to preservation issues. Normative mortuary practices sometimes do not even include complete burials or cemeteries (36), and in the cases of widespread excarnation or other kinds of nonburial, reliable data will always be absent. While cremations, which significantly transform and fragment skeletal remains, were rather rare in the Neolithic, collective burials were not. This practice, over time, often led to the disarticulation and disassociation of skeletal individuals. Coupled with the often fragmentary nature of the bone remains themselves, the result is a lack of reasonably complete “individual” skeletons that could have been assessed for signs of trauma. In many cases, every skull fragment has to be assessed on its own, leading to a lack of precise demographic and other interrelated data. As a consequence, in times and regions where a collective burial rite was practised, trauma data will always be less detailed compared with communities that practised standard inhumation. For the latter, single burials were usually the norm, but carefully arranged graves containing more than one person also frequently occur. Single burials of injured individuals tell us very little about the context in which a traumatic injury was sustained and while there is little doubt that the targeted killing of individuals is likely to have occurred, accidents cannot be ruled out completely in some cases.

In contrast, double, triple, or multiple burials most likely indicate that several people died at the same time. This could have had a variety of causes, including lethal violence. Numerous multiple burials from the period show clear skeletal evidence of violent death (e.g., refs. (37) and (38)). Depending on their size, they can indicate conflict between individuals, between smaller groups, and even whole communities. The more individuals died concurrently, the more likely it is that these deaths may be related to actual organized warfare or other violence-related group activities that involved larger sections of or even entire communities. In these planned, premeditated attacks, every member of the target group could become a victim of lethal violence (cf (39)). High numbers of children and women among the dead from conflict-related mass and multiple burials demonstrate that group membership was the reason enough to be killed. This practice of social substitution, where every individual of the opposing group was interchangeable as a legitimate victim, seems to be one of the key characteristics of Neolithic mass violence (40).

## From Site Studies to Population Perspectives: Quantifying Violence

As stated, many skeletal samples from Northwestern Europe present particular challenges for quantifying violence due to collective burial, complex multistage mortuary treatments, and their results (e.g., extensive commingling, disarticulation, and fragmentation). In consequence, assemblages comprised of hundreds or thousands of bone fragments commonly return minimum number of individuals (MNI) estimates in just single or double figures (e.g., refs. (41)–42)). These aspects have particular implications for the analysis of violence-related injuries, as it is frequently impossible to tell whether or not an individual sustained injuries in more than one part of the body. Demographic assessments from individual bones or fragments are often rendered impossible beyond the observation that an individual was an “adult.” In such assemblages, the most reliable indicator of prevalence rates is generally the cranium, as it has high potential for discerning a reasonable MNI and was frequently targeted in violent assaults. Consequently, the most accurate prevalence rates available are those for cranial trauma. As it is not possible within the scope of the article to discuss all of Neolithic Europe in detail, some comparatively well-examined regions have been selected here as illustrative examples.

The British Isles is a region that particularly exemplifies the above issues. Schulting and Wysocki’s (23) study of 350 Neolithic crania from Britain (ca 4300–2000 BC) identified 30 instances of trauma consistent with violence (21 healed, 9 unhealed) comprising 8.57% of the sample. Additional examples that have been examined or published since have added a further 106 crania, 26 of which bore injuries (11 healed, 14 unhealed), bringing the revised total to 56/456 (12.28%). It is frequently more challenging to attribute causation to injuries affecting other parts of the skeleton and therefore often impossible to distinguish violence-related trauma from accidental injuries to infracranial bones. Notwithstanding this point, 17 instances of infracranial trauma consistent with violence have been identified among British samples (7 healed, 10 unhealed). Among the latter, all of the unhealed examples were projectile injuries exhibiting embedded or associated arrowheads. While these infracranial traumata add to the emerging picture of endemic violence, it is not possible to incorporate them in general prevalence figures, due to the aforementioned challenges in assessing the overall number of individuals represented.

**Fig. 4.**
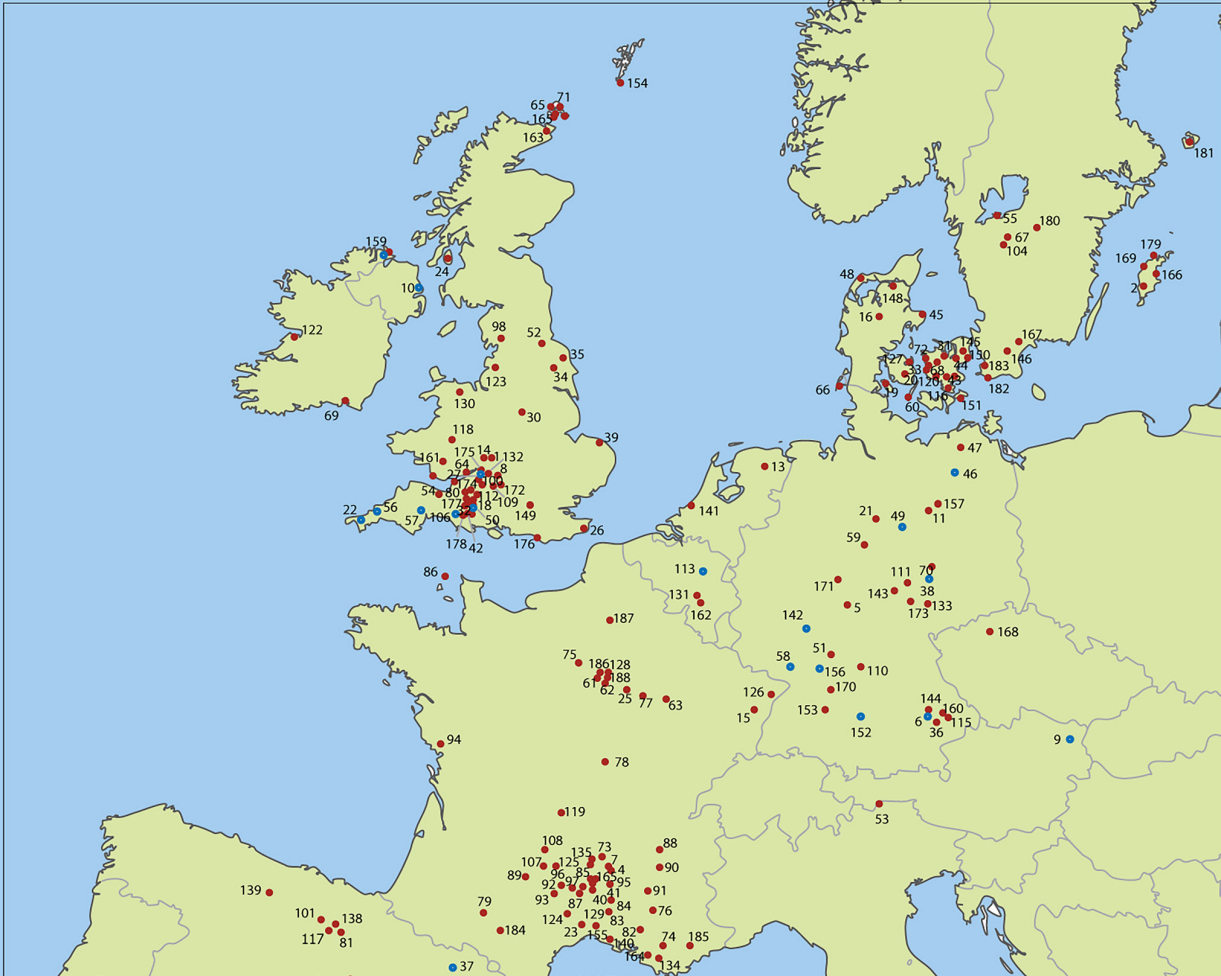
Map of northwestern Europe showing archaeological sites with violence-related injuries in Neolithic skeletal remains (red) and settlements/enclosures/mass fatality sites with evidence for collective violence (blue). See *SI Appendix* for site names and additional references.

British and Irish examples generally occur in low numbers, with often only one or two individuals within an assemblage exhibiting violence-related injuries. Mass burials relating to larger-scale episodes of violence are not (yet) known from Neolithic Britain or Ireland, although several of the identified projectile wounds were among individuals associated with defended sites showing evidence for attack. Seven such sites have been identified, six of which are within the southwest of England. Where enclosures show evidence of such attacks, it is clear that at least some instances of conflict in Britain involved larger groups (43–45) (Fig. 4).

In contrast, the agricultural lifeway reached southern Scandinavia even later than it did Neolithic Britain at ca. 4000 BC and introduces the Neolithic period to the region (4000–1700 BC). Advances in DNA analysis have rewritten the narrative of the Neolithic here (46), highlighting a complex demographic history with an agricultural economy being established as a result of a demic diffusion. From the earliest phase (4000–3300 BC), human skeletal remains derive from wetland contexts and graves (mainly dolmens). The wetland finds were recently summarized by Sjögren et al. (47), with five individuals with traumatic injuries, and four with cords around the neck, one of which also displayed a traumatic injury. The general prevalence of violence is difficult to deduce from these remains as they are biased toward younger age groups. Most of the human bones from the first agriculturalists (Funnel Beaker Culture 4000–2900 BC) have been uncovered from megalithic tombs, where dolmens are generally dated to the Early Neolithic and the larger passage tombs to the early Middle Neolithic (3300–2900 BC).

Specifically, cranial trauma has been studied by Fibiger et al. (3), mainly from megalithic tombs. In all, 16.9% (n = 261) of the Danish sample and 9.4% (n = 117) of the Swedish sample have cranial trauma, with 4.6% and 2.6% unhealed trauma, respectively. Of twelve previously reported Neolithic trepanations from Denmark, eight were reassessed as traumatic in origin (48). In fact, some of the allegedly trepanned skulls from Sweden have been reassessed as traumatic in origin as well (3, 49), highlighting again the challenges of older osteological assessments and more recent rediagnoses.

Around 3200 BC, there is a shift in material culture marking a partial return of a hunter-gatherer adaptation (i.e., Pitted Ware Culture, PWC, and 3200–2300 BC), a very different picture from the rest of Europe. The PWC has a geographical distribution that embraces the coastlines of southern Norway, western, southern and eastern Sweden, as well as the islands in the Baltic proper. From a genetic point of view, the results point toward an affinity with western Mesolithic hunter-gatherers (46). Trauma in the adult sample from Gotland in the Central Baltic Sea amounts to 9.2% (13/141). Of the 116 adult individuals from PWC graves from Gotland, 12 (10.4%) have cranial trauma, comprising four females (4/53, one fatal, others healed) and eight males (8/63, all healed). These statistics deviate from those of Ahlström and Molnar (49) as sample size has increased. Cut marks have also been used to infer cannibalism based on a PWC context at Jettböle, Åland (50). Around 2800 BC, there was yet another major demographic shift as continental Corded Ware groups, locally designated as the Battle Axe Culture (BAC) or Single Grave Culture (SGC), appeared in Scandinavia. There are fewer skeletal remains from this group, but traumatic injuries are present at Viby, Östra Torp, and Tygelsjö (3, 51). In summary, the skeletal evidence from the Neolithic period in Southern Scandinavia suggests endemic violence was present, although no evidence of mass violence has been found to date.

Enclosures, causewayed (c. 3300–3100 BC) and palisaded (c. 2900–2600 BC), are known from southwestern Scandinavia, mainly Denmark and Scania (52). Interpretations regarding these structures have varied, from religious activities to corrals for cattle. Another plausible explanation is that they represent fortifications (53, 54). The appearance of PWC correlates with the first construction of enclosures, BAC with the second, possibly signifying social unrest. It is, however, exceedingly difficult to assess whether or not trauma was caused by conflicts that may be portrayed as intragroup, intergroup, or interindividual. Iversen (55) presented a functional assessment of tanged projectiles associated with the PWC and concluded that the long, narrow, and slender C-type, without barbs though, was used for war-like purposes. This projectile is relatively common in megalithic tombs, possibly introduced into the chambers within the bodies of victims, which is also seen in other regions of Europe (15). Tanged points are also present in megalithic tombs from Falbygden, Sweden, but are not as systematically studied as the finds from Denmark (56). None have been found embedded in bone, and the only example of a tanged point embedded in bone would rather suggest intragroup violence. Individual D from the Gjerrild (Jutland) stone cist, associated with the SGC, has a type D tanged point embedded in the breastbone (57). This type of projectile has typically been associated with the SGC (55). Similarly, the cranium from Tygelsjö, Scania, with an antler pickaxe firmly embedded in the parietal bone, cannot be used to discuss intergroup violence as both the PWC and BAC used these implements.

### Intergroup Mass Violence in Early Neolithic Central Europe.

Episodes of extreme mass violence during the Neolithic are now well known and their analysis is almost guaranteed to receive widespread attention (21, 38, 58). This certainly biases general discussions in favor of these special sites, while other, much less spectacular, sites receive less consideration. While injuries potentially caused by interpersonal violence have accompanied humankind likely from the very beginning (59), the evidence for mass violence between defined communities in the Neolithic is noteworthy. In part, this impression is likely linked to the more extensive skeletal record compared to the preceding Palaeolithic and Mesolithic. With a settled lifestyle, one of the traditional hallmarks of the Neolithic, also came more widespread permanent burial sites that, over time, could incorporate several hundred individuals. For some (later) Neolithic cultures, it is evident that people with serious trauma were not distinguished by differential burial rites or spaces (60), indicating that suffering violent injuries at the hands of others was an accepted and possibly expected part of life.

In other phases of the Neolithic, serious skeletal trauma is unexpectedly rare. This is the case for the very first farmers in Central Europe, the LBK, at least when looking at the more commonplace burial record. Here, individuals who bear the signs of potentially lethal violent injuries are infrequent and only few convincing examples have been described to date (e.g., ref. (61)). Given the number of currently known LBK individuals from large parts of Central Europe (ca. 3,000), this might indicate that people who died violently or were visibly affected by violence during their lifetimes were usually not included in more conventional burial sites. In contrast, the LBK is also known for the repeated occurrence of actual mass graves and similar sites containing the bodies of dozens of individuals with extensive lethal trauma (21, 22, 62, 63). As these have been found in different parts of the LBK sphere, the respective mass violence events were not one-off chance occurrences, but part of a behavioral pattern toward the end of the LBK sequence, just before 5000 cal BC. It is commonly thought that whole local communities may have been wiped out during massacres then (64). As a consequence of this highly visible and largely indiscriminate lethal violence, sometimes including torture or mutilation (21), these mass violence sites occupy a central position in the discussions of Neolithic conflict and warfare. Not infrequently, several other unusual or atypical burial and deposition sites are also subsumed under the umbrella of massacres, even if they are very likely to have been the result of very different processes (65).

While Neolithic massacres undoubtedly happened, their true significance and frequency are still disputed, as are the criteria for giving these individuals a formal or permanent burial near their communities (66). The LBK did not invent lethal group violence, as earlier examples are known (e.g., refs. (67) and (68)), but their repeated and likely patterned utilization of extreme lethal violence is notable. At the same time, during most of the LBK period, such mass violence was absent, at least as far as we can determine this archaeologically. The question remains whether the massacres apparently clustering at the end of the LBK are truly representative of the culture as a whole, or if they are an exception, caused by a unique cooccurrence of natural and societal factors that repeatedly drove communities to seek extreme measures. Comparative research shows that such massacres occur within a particular context and are often the result of lengthy societal processes and increased power imbalances between different groups. The victims tend to be dehumanized by the aggressors, which in the known examples is expressed by denying them meaningful individual burials and by potentially mutilating their corpses (30). There is some evidence that within the first farming communities of Central Europe, the conditions for the escalation of conflicts were increasingly met with time, including the development of social differentiation, unequal access to resources, and definitions of self and group identity to the detriment of others (e.g., (69)). Mass violence, therefore, appears to be the occasional, extreme end point in a network of longer lasting intercommunity relations, chosen toward the end of the LBK by multiple, independent communities. The killings themselves were most likely carried out by ordinary people with standard Neolithic weapon tools. Only toward the latest stages of the Neolithic, the concept of specialized warriorhood slowly started to emerge. The perpetrators of violence might have become more clearly defined, while lethal raids and indiscriminate massacres still occurred, as multiple examples show (e.g., refs. (38, 70, and 71)). In the following Bronze Age, the face of conflict changed even further, to include specialized weapons of violence and large-scale battles likely fought by dedicated warriors (72).

### Motivations for Violence.

In seeking to characterize the motivations behind the violent acts perpetrated during the Neolithic, a question of key importance is whether violent interactions occurred within or between social groups. It is likely that the datasets that survive contain examples of both, although in a society characterized by relatively small groups, the loss of even a small number of individuals could harbor severe negative consequences in terms of adaptive fitness and long-term survival. Consequently, it is reasonable to suggest that nonlethal assaults are more likely, on balance, to relate to violence within communities, while at least the majority of lethal injuries more likely occurred between groups (73). This latter suggestion might raise objections over the problem of discerning intent. A blow struck in anger might result in an individual’s death even if that was not the premeditated intention of the assailant. While this is certainly true, many of the injuries observed on Neolithic remains can only have resulted from substantial impacts inflicted with implements that carry a high degree of lethality. A full force blow to the head with a stone axe is more likely than not to cause death. The same is true of projectile injuries, which entail not only lethal intent but also advanced planning and do not lend themselves to close-contact scenarios involving spontaneous outbursts of anger.

On the surface, resolving the question of whether or not lethal assaults took place in the context of “war” as opposed to disputes between individuals might seem simply a question of numbers. While the mass burials necessitate hostilities between groups, these comprise a minority of the overall sample sites. The great majority of examples of Neolithic violence involve only one or two injured individuals. A key feature of the arguments proffered by proponents on opposing sides of the debate over the existence of war in prehistory is the view taken over such “individual” instances. Such examples can easily be attributed as “homicides,” with examples involving multiple individuals similarly dismissed as “feuding” between small groups (74). Feuding is essentially a situation where two groups regard themselves as being “at war” with each other in terms of recognizing a state of ongoing mutual hostility, in which any group member can represent the whole and is liable to be considered a target. The classification of “homicide” may be problematic in this context as it derives from modern perceptions that focus on behavior at the expense of motivation.

Contrary to such notions, war among prestate societies may have followed prescribed and socially sanctioned sets of “rules” and need not entail the complete annihilation of an opposing group as the ultimate objective. A situation of reciprocal raiding where participants (almost exclusively young adult males) elevate their status through killing or capturing enemies might result in raiding bands consisting of perhaps a dozen individuals ambushing and killing just one or two members of an “opposing” community. In functioning as a means to climb the social hierarchy, such acts of “conflict” can in fact relate more to “intragroup” tensions than enmities between different communities. Where perceptions of external threats of violence persist over time, these might be discerned archaeologically in the form of communal constructions. The creation and maintenance of defensible structures might seem an obvious indication of such, especially if signs of attack are in evidence (43, 44). However, the coappearance of monumentality with domestication, the *sine qua non* of the Neolithic, may also indicate the prevalence of new and ongoing social tensions, as a material expression of intensified territoriality.

During the latter half of the 20th century, Neolithic societies were generally regarded as essentially unstratified, with limited variation in material wealth and low levels of skill specialization. Emergent polities tended to be small scale, with competition for resources limited, as populations remained small and relatively scattered (75–77). This latter view was intertwined with a general view of the Neolithic as a relatively peaceful time in which the political and economic conditions associated with the forms of warfare seen in later periods had yet to come into effect. More recently, the underlying assumptions regarding the motivations for intergroup violence on which such views are based have been challenged (25, 78. They are based on a view that “war” is a behavior peculiar to complex societies with an attendant expectation that it simply did not occur prior to proto-state formation, metallurgy, and increasing wealth disparities (74). In addition, while the observation that there is a positive relationship between social inequality and violence is well evidenced (79, 80), assumptions of there being only minimal economic disparity among Neolithic groups are open to challenge.

While lethal and sublethal violence were clearly a feature of life prior to domestication, there is unequivocal evidence of significant change during the Neolithic in terms of the scale, frequency, and tactics involved in violent interactions, when compared to the Mesolithic and earlier periods (81, 82). Prior to domestication, episodes of massed violence as indicated by multiple burials and attacks on fortified structures during the Neolithic are neither in evidence nor were they possible due to the small-scale, scattered, and more mobile nature of forager populations. As stated, direct comparisons of the prevalence of violence-related assaults are not currently feasible, although it is salient to note that in regions where the condition of Neolithic skeletal remains is closest to the situation seen among Mesolithic samples, in being generally fragmentary, disarticulated, and poorly preserved, such as Britain, the rates of trauma remain markedly higher postdomestication. The assertion that violence was more frequent among farmers than in earlier periods is therefore argued to be a real distinction rather than simply an artifact of preservation. Developments in scale, frequency, and tactics related to violence may in fact be rooted in changes to the economic basis of societies at this time and related effects on social relations. Forager group sizes tend to be constrained by the carrying capacity of the land and the practicalities of transporting small children. Neolithic lifeways favor larger families and led to the Neolithic Demographic Transition (8), an effective population explosion in which societies became unbalanced toward younger individuals. While “successful” foragers can only share the benefits of their efforts in the short term and with a few individuals, successful farmers can accumulate material wealth in the form of cleared land and livestock that both permit and promote ever larger family sizes. These new forms of wealth were also heritable, meaning that emerging wealth disparities could grow wider across multiple generations. The emergence of “wealthy” individuals, especially in more pastoralist groups, will also have created conditions that favored polygamy––some individual males were now able to support more than one spouse. This change would further increase inequality by producing powerful patriarchs at the head of increasingly large families while also disenfranchising other males who might be unable to marry. The former hypothesis appears to be borne out by the recent aDNA study of remains from Hazleton North chambered tomb, southwest England (83), where a single male progenitor had reproduced with four women to produce a five-generation family, with female exogamy. The combination of material, social, and reproductive inequalities created by the conditions arising from domestication contrasts with former egalitarian perceptions of the Neolithic. These new inequalities would be sufficient to account for both the motivations behind the forms of intergroup violence now prevalent and also the form of such interactions with raiding and the abduction of women as apparent among repeated mass burials, now a recurring feature of intergroup hostilities.

A recent comparative study of Early Neolithic LBK mass fatality sites from Central Europe confirmed that many sites seem to have a lack of certain demographic components (39). Men, women, and children apparently were legitimate targets during mass violence events and were frequently killed in them. At the same time, there is a comparative dearth of younger women at all the three currently known LBK massacre sites where it is presumed that a complete settlement was eradicated (Talheim, Asparn/Schletz, and Schöneck-Kilianstädten). At the site of Schöneck-Kilianstädten at least, this demographic gap also extends to adolescents, who were absent among the victims in the mass grave. The most likely explanation is that these were indeed selectively taken captive as possible spoils of warfare between groups, as is also known from ethnographic examples (84). Possibly, they were integrated into the attacker’s own groups, but their social standing and actual fate are, so far, unknown (39). While abduction might have been a part of everyday Neolithic life, necessitating certain precautions on the part of the most likely victims and their daily routines, such practices only become clearly visible archaeologically in mass fatality events that could have affected everyone within a raided settlement. Only in the aftermath of such a cataclysmic mass violence event, i.e., the resulting mass or multiple burials, can we ascertain a lack of a specific demographic group with the necessary fine-grained chronological resolution, as all were buried at the same time, as a snapshot of a Neolithic community. Burial in a conventional cemetery or collective grave used over longer periods of time just cannot provide the same chronological resolution, rendering the selective capturing of people invisible (66).

## Conclusions

Ongoing study of prehistoric skeletal assemblages from a variety of periods continues to improve the scale and resolution at which we can recognize and understand past acts of violence. Consideration of patterns and prevalence at a broad regional and diachronic scale, as presented in the current study, determines that for Northwestern Europe at least, the conception of the “peaceful Neolithic” is dead. While violent hostilities between groups were not an innovation in themselves, the practice, scale, and prevalence of human violence appear to have undergone dramatic and lasting changes during this period. It is likely that war developed greater meaning and complexity as a social strategy, with implications for both individuals and groups to advance themselves at the expense of others, setting a pattern that was to persist. The extent to which prehistoric communities were more or less prone to violence than the more complex societies that superseded them has been central to debates regarding the overall character of both prehistoric life and human nature in general since the 17th century. In recent times, Stephen Pinker’s (85) widely read and frequently cited but also criticized work (86) took the position that aggregate levels of violence have been in decline for centuries. The predominantly historical sources on which Pinker’s thesis was based become progressively less distinct with increasing distance from the present, and his study virtually omits the Neolithic skeletal record in his assertions (87). The current study and other recent publications regarding the Neolithic present a strong case for this period possibly representing a high watermark of human violence. Developments regarding the conceptualization, frequency, and form of organized conflict may in fact be a legacy of the Neolithic that ranks among the greatest societal impacts of the shift to domestication. At the same time, as this study (and indeed this special edition) show, there is diversity across space and time, reenforcing the complexity and contextual qualities of human behavior.

## Supplementary Material

Appendix 01 (PDF)

## Data Availability

All study data are included in the article and/or *SI Appendix*.
