# Supplementary material for: Conflict, violence, and warfare among early farmers in Northwestern Europe
Source: Proc Natl Acad Sci U S A. 2023 Jan 17;120(4):e2209481119. doi: 10.1073/pnas.2209481119 (PMC9942812; doi:10.1073/pnas.2209481119)
Supplement: Supplementary file 1 — Appendix 01 (PDF) [file pnas.2209481119.sapp.pdf]

Supplementary Material

Sites listed on Map (Figure 4)

No.	Site Name	Country	Reference	Ref. No.
1	Adlestrop	England	Smith (2005)	90
2	Ajvide	Sweden	Ahlström & Molnar (2012)	51
3	Alba Iulia-Lumea Nouã	Romania	Gligor *et al*. (2018)	91
4	Almieres, Saint Rome de Dolan	France	Guilaine & Zammit (2001)	92
5	Altendorf	Germany	Fibiger (2012)	19
6	Altheim	Germany	Petrasch (1999)	93
7	Aragon, Le Massegros	France	Guilaine & Zammit (2001)	92
8	Ascott under Wychwood	England	Benson & Whittle (2006)	94
9	Asparn-Schletz	Austria	Teschler-Nicola (2012)	64
10	Ballyharry	Ireland	Moore (2004)	95
11	Barleben	Germany	Fibiger (2009)	96
13	Beilerstroom Valley	Netherlands	v. d. Sanden & ter Schegget (2015)	97
14	Belas Knap	England	Schulting & Wysocki (2005)	23
15	Bergheim	France	Chenal *et al*. (2015)	98
16	Bidstrup	Denmark	Fibiger *et al.* (2013)	3
17	Bòvila Madurell	Spain	Beyneix 2012	99
18	Boles Barrow	England	Smith & Brickley (2009)	100
19	Bolkilde I	Denmark	Bennike *et al.* (1986)	101
20	Borreby XIV	Denmark	Fibiger *et al.* (2013)	3
21	Bredelem	Germany	Fibiger *et al.* (2013)	3
22	Carn Brea	England	Mercer (1999)	46
23	Castelnau-le-Lez	France	Guilaine & Zammit (2001)	92
24	Clachaig, Isle of Arran	Scotland	Smith (2017)	25
25	Coizard	France	Guilaine & Zammit (2001)	92
26	Coldrum	England	Schulting & Wysocki (2005)	23
27	Crickley Hill	England	Mercer (1999)	46
29	Cueva Honda	Spain	Jimenez-Brobeil *et al.* (2009)	102
30	Dinnington	England	Schulting & Wysocki (2005)	23
31	Døringe I	Denmark	Bennike (1985)	103
32	Dorsetshire Long Barrow	England	Schulting & Wysocki (2005)	23
33	Dræby	Denmark	Fibiger *et al.* (2013)	3
34	Duggleby Howe	England	Gibson & Ogden (2008)	104
35	Ebberston	England	Schulting & Wysocki (2005)	23
36	Eichendorf	Germany	Fibiger (2009)	96
37	Els Trocs	Spain	Alt *et al.* (2020)	105
38	Eulau	Germany	Meyer *et al*. (2009)	37
39	Fengate	England	Pryor (1976)	106
40	Fontcagarelle	France	Guilaine & Zammit (2001)	92
41	Font-Rial	France	Guilaine & Zammit (2001)	92
42	Fussels Lodge	England	Smith (2013)	4
43	Gammellung	Denmark	Bennike (1985)	103
44	Ganløse	Denmark	Fibiger *et al.* (2013)	3
45	Gjerrild	Denmark	Bennike (1985)	103
46	Goldberg	Germany	Fibiger (2009)	3
47	Gross Upahl	Germany	Lidke (2012)	107
48	Grydehoj II	Denmark	Fibiger *et al.* (2013)	3
49	Halberstadt	Germany	Meyer *et al.* (2018)	39
50	Hambledon Hill	England	Mercer & Healy (2008)	108
51	Handschuhsheim	Germany	Wahl & Höhn (1988)	109
52	Hartlepool	England	Schulting & Wysocki (2005)	23

53	Hauslabjoch (Ötzi)	Austria	Pernter *et al.* (2007)	110
54	Hay Wood Cave	England	Schulting (2012)	111
55	Hellekis	Sweden	Retzius (1900)	112
56	Helman Tor	England	Mercer (1999)	46
57	Hembury	England	Mercer (1999)	46
58	Herxheim	Germany	Orschiedt *et al.* (2003)	113
59	Hildesheim	Germany	Schutkowski *et al.* (1996)	14
60	Hulbjerg	Denmark	Bennike (1985)	103
61	Hypogeum 1, Oyes	France	Guilaine & Zammit (2001)	92
62	Hypogeum 2, Oyes	France	Guilaine & Zammit (2001)	92
63	Hypogeum II, Villevenard	France	Guilaine & Zammit (2001)	92
64	Ifton Quarry Cave	England	Schulting (2020)	114
65	Isbister	Scotland (Orkney)	Lawrence (2012)	115
66	Juelsberg	Denmark	Fibiger *et al.* (2013)	3
67	Karleby	Sweden	Fibiger *et al.* (2013)	3
68	Kelderød	Denmark	Bennike (1985)	103
69	Kilgreany	Ireland	Fibiger (2016)	116
70	Kitzen	Germany	Fibiger (2009)	96
71	Knowe of Rowiegar	Scotland (Orkney)	Smith & Brickley (2009)	100
72	Kyndelose IX	Denmark	Fibiger *et al.* (2013)	3
73	L'Aumede	France	Guilaine & Zammit (2001)	92
74	La Fare	France	Guilaine & Zammit (2001)	92
75	La Ferme Duport	France	Guilaine & Zammit (2001)	92
76	La Lave	France	Guilaine & Zammit (2001)	92
77	La Pierre Michelot	France	Guilaine & Zammit (2001)	92
78	La Pierre Plate	France	Guilaine & Zammit (2001)	92
79	La Tourasse	France	Guilaine & Zammit (2001)	92
80	Lanhill	England	Smith (2013)	4
81	Las Yurdinas II	Spain	Fernández-Crespo (2017)	117
82	Le Capitaine	France	Guilaine & Zammit (2001)	92
83	Le Castellet	France	Guilaine & Zammit (2001)	92
84	Le Chemin-de-Fer	France	Guilaine & Zammit (2001)	92
85	Le Crespin	France	Guilaine & Zammit (2001)	92
86	Le Dehus	Guernsey	Schulting *et al.* (2010)	118
87	Le Monna	France	Guilaine & Zammit (2001)	92
88	Le Pas-de-Jouille	France	Guilaine & Zammit (2001)	92
89	Le Sot-de-la -Lavogne	France	Guilaine & Zammit (2001)	92
90	Les Baumes-Chaudes	France	Guilaine & Zammit (2001)	92
91	Les Boileau	France	Guilaine & Zammit (2001)	92
92	Les Caires	France	Guilaine & Zammit (2001)	92
93	Les Cascades	France	Guilaine & Zammit (2001)	92
94	Les Châtelliers du Vieil-Auzay	France	Guilaine & Zammit (2001)	92
95	Les Crottes	France	Guilaine & Zammit (2001)	92
96	Les Gaches	France	Guilaine & Zammit (2001)	92
97	Les Treilles	France	Guilaine & Zammit (2001)	92
98	Lesser Kelco Cave	England	Leach (2008)	119
100	Littleton Drew	England	Smith & Brickley (2009)	100
101	Longar	Spain	Marquez *et al.* (2005)	120
104	Luttra	Sweden	Fibiger *et al.* (2013)	3
106	Maiden Castle	England	Mercer (1999)	46
107	Mauray	France	Guilaine & Zammit (2001)	92
108	Maymac	France	Guilaine & Zammit (2001)	92
109	Millbarrow	England	Schulting & Wysocki (2005)	23
110	Neckargartach	Germany	Fibiger (2009)	96

111	Niederbösa	Germany	Fibiger (2009)	96
112	Norton Bavant	England	Schulting & Wysocki (2005)	23
113	Oleye	Belgium	Lodewijckx (2009)	121
115	Otzing	Germany	Fibiger (2009)	96
116	Over Vindinge	Denmark	Fibiger *et al.* (2013)	3
117	Peña de Marañón	Spain	Fernández-Crespo (2016)	122
118	Penywyrlod	Wales	Wysocki & Whittle (2000)	123
119	Pontcharraud	France	Guilaine & Zammit (2001)	92
120	Porsmosen	Denmark	Bennike (1985)	103
122	Poulnabrone	Ireland	Schulting & Wysocki (2005)	23
123	Preston Docks	England	Schulting & Wysocki (2005)	23
124	Previnquieres	France	Guilaine & Zammit (2001)	92
125	Puechcamp	France	Guilaine & Zammit (2001)	92
126	Quatzenheim	France	Guilaine & Zammit (2001)	92
127	Rævehøj	Denmark	Fibiger *et al.* (2013)	3
128	Razet	France	Guilaine & Zammit (2001)	92
129	Rec de Las Balmos	France	Guilaine & Zammit (2001)	92
130	Rhos Ddigre	Wales	Schulting (2012)	111
131	Rochers de Freyr (Abri du Pape)	Belgium	Polet & Dutour (2007)	124
132	Rodmarton	England	Smith & Brickley (2009)	100
133	Salzmünde	Germany	Meyer *et al.* (2013)	125
134	Saint-Clair	France	Guilaine & Zammit (2001)	92
135	Saint-Enimie	France	Guilaine & Zammit (2001)	92
138	San Juan ante Portam Latinam	Spain	Vegas *et al.* (2012)	15
139	San Quirce des Vallés	Spain	Armendariz *et al.* (1994)	126
140	Sargel, Saint-Rome de Cemon	France	Guilaine & Zammit (2001)	92
141	Schipluiden	Netherlands	Smits (2012)	127
142	Schöneck-Kilianstädten	Germany	Meyer *et al.* (2015)	21
143	Schönstedt	Germany	Fibiger (2009)	96
144	Sengkofen	Germany	Fibiger *et al.* (2013)	3
145	Sigersdal	Denmark	Bennike (1985)	103
146	Skepparslöv	Sweden	Fibiger *et al.* (2013)	3
148	Sønderup III	Denmark	Fibiger *et al.* (2013)	3
149	Staines	England	Schulting (2012)	111
150	Steenstrupp	Denmark	Fibiger *et al.* (2013)	3
151	Stege	Denmark	Fibiger *et al.* (2013)	3
152	Stetten ob Lontal	Germany	Conard *et al.* (2004)	128
153	Stuttgart-Mühlhausen	Germany	Fibiger (2009)	96
154	Sumburgh	Scotland (Shetland)	Walsh *et al.* (2011)	129
155	Suquet-Coucolieres	France	Guilaine & Zammit (2001)	92
156	Talheim	Germany	Wahl & König (1987)	130
157	Tangermünde	Germany	Fibiger (2009)	96
158	Terrevaine, La Ciotat	France	Guilaine & Zammit (2001)	92
159	Thornhill	Ireland	Moore (2004)	95
160	Tiefbrunn	Germany	Fibiger (2009)	96
161	Tinkinswood	Wales	Schulting & Wysocki (2005)	23
162	Trou Rosette	Belgium	Polet *et al.* (1996)	131
163	Tulloch of Assery	Scotland	Schulting & Wysocki (2005)	23
164	Tumulus de Gendarme	France	Guilaine & Zammit (2001)	92
165	Unstan	Scotland (Orkney)	Ahlström & Molnar (2012)	51
166	Västerbjers	Sweden	Ahlström & Molnar (2012)	51
167	Viby	Sweden	Fibiger *et al.* (2013)	3
168	Vikletice	Czech Republic	Shbat *et al.* (2009)	132
169	Visby	Sweden	Ahlström & Molnar (2012)	51

170	Waiblingen	Germany	Fibiger (2009)	96
171	Warburg	Germany	Fibiger (2012)	19
172	Waylands Smithy	England	Whittle *et al*. (2007)	133
173	Weimar	Germany	Fibiger (2009)	96
174	West Kennet	England	Smith & Brickley (2009)	100
175	West Tump	England	Smith & Brickley (2009)	100
176	Whitehawk Hill	England	Schulting (2012)	111
177	Winterbourne Monkton	England	Schulting & Wysocki (2005)	23
178	Wor Barrow	England	Allen *et al.* (2016)	134
179	Ihre	Sweden	Ahlström & Molnar (2012)	51
180	Alvastra	Sweden	During & Nilsson (1991)	135
181	Jettböle	Sweden	Nunez (1995)	136
182	Östra Torp	Sweden	Fibiger *et al*. (2013)	3
183	Tygelsjö	Sweden	Ahlström & Molnar (2012)	51
184	Montfort-sur-Lizier	France	Guilaine & Zammit (2001)	92
185	Fontbregoua Cave	France	Guilaine & Zammit (2001)	92
186	La Cimitière des Anglais	France	Guilaine & Zammit (2001)	92
187	Suzoy	France	Guilaine & Zammit (2001)	92
188	Hypogeum II, Oyes	France	Guilaine & Zammit (2001)	92

Additional References

90. M. J. Smith, *Picking up the Pieces: an Analysis of Cotswold Severn Funerary Practices via Re-examination of Human Skeletal Material from Selected Monuments*, PhD Thesis (University of Birmingham, UK, 2005).

91. M. Gligor, A. D. Soficaru, A. Fetcu, "Cranial fractures in 2005 early Eneolithic multiple burial From Alba Iulia-*Lumea Nouă* (Romania)" in *Archaeology of Women. Mortuary Practices and Bioarchaeological Reconstruction*, M. Gligor, A. Soficaru, Eds. (Editura Mega, 2018), pp. 27–76.

92. J. Guilaine, J. Zammit, *Le Sentier de la guerre. Visages de la violence préhistorique* (Seuil, 2001).

93. J. Petrasch, Mord und Krieg in der Bandkeramik. *Archäologisches Korrespondenzblatt* **29**, 505–516 (1999).

94. D. Benson, A. Whittle, *Building Memories: The Neolithic Cotswold Long Barrow at Ascott-Under-Wychwood* (Oxbow, 2006).

95. D. G. Moore, "Hostilities in early Ireland: Trouble with the new neighbours –the evidence from Ballyharry, County Antrim" in *From Sickles to Circles: Britain and Ireland at the Time of Stonehenge*, A. Gibson, A. Sheridan, Eds. (Tempus, 2004), pp. 142–154.

96. L. Fibiger, *Heading for Trouble: Skeletal Evidence for Interpersonal Violence in Neolithic Northwest Europe*, DPhil Thesis (University of Oxford, 2009).

97. W. van der Sanden, M. ter Schegget, "Frühe Gewalt in den Niederlanden. Der Mann aus dem Beilerstroom" in *Krieg – Eine archäologische Spurensuche*, H. Meller, M. Schefzik, Eds. (Theiss, 2015), pp. 145–147.

98. F. Chenal, B. Perrin, H. Barrand-Emam, B. Boulestin, A farewell to arms: A deposit of human limbs and bodies at Bergheim, France, *c.* 4000 BC. *Antiquity* **89**, 1313–1330 (2015).

99. A. Beyneix, "Neolithic violence in France: an overview" in *Sticks, Stones, and Broken Bones. Neolithic Violence in a European Perspective*, R. J. Schulting, L. Fibiger, Eds. (Oxford University Press, 2012), pp. 207–221.

100. M. Smith, M. Brickley (Eds.) *People of the Long Barrows: Life, Death and Burial in the Earlier Neolithic* (The History Press, 2009).

101. P. Bennike, K Ebbesen, L.B. Jørgensen, Two Early Neolithic skeletons from Bolkilde bog, Denmark. *Antiquity* **60**, 199–209 (1986).

102. S. Jimenez-Brobeil, P. du Souich, I. Al Oumaoui, Possible relationship of cranial traumatic injuries with violence in the south-east Iberian Peninsula from the Neolithic to the Bronze Age. *Am. J. Phys. Anthropol*. **140**, 465–475 (2009).

103. P. Bennike, *Palaeopathology of Danish Skeletons* (Akademisk Forlag, 1985).

104. A. M. Gibson, A. R. Ogden, Duggleby Howe, Burial J and the Eastern Yorkshire Club Scene. *Yorkshire Archaeological Journal* **80**, 1–13 (2008).

105. K. W. Alt et al., A massacre of early Neolithic farmers in the high Pyrenees at Els Trocs, Spain. *Scientific Reports* 10, 2131 (2020).

106. F. Pryor, A Neolithic multiple burial from Fengate, Peterborough. *Antiquity* **50**, 232–233 (1976).

107. G. Lidke, "Violence in the Single Grave culture of northern Germany?" in *Sticks, Stones, and Broken Bones. Neolithic Violence in a European Perspective*, R. J. Schulting, L. Fibiger, Eds. (Oxford University Press, 2012), pp. 139–150.

108. R. Mercer, F. Healy, *Hambledon Hill, Dorset, England* (English Heritage, 2008).

109. J. Wahl, B. Höhn, Eine Mehrfachbestattung der Michelsberger Kultur aus Heidelberg-Handschuhsheim, Rhein-Neckar-Kreis. *Fundberichte aus Baden-Württemberg* **13,** 123–198 (1988).

110. P. Pernter, P. Gostner, E. Egarter Vigl, F. J. Rühli, Radiologic proof for the Ice-man's cause of death (ca. 5,300 BP). *Journal of Archaeological Science* **34**, 1784–1786 (2007).

111. R. J. Schulting, "Skeletal evidence for interpersonal violence beyond mortuary monuments in southern Britain" in *Sticks, Stones, and Broken Bones. Neolithic Violence in a European Perspective*, R. J. Schulting, L. Fibiger, Eds. (Oxford University Press, 2012), pp. 223–248.

112. G. Retzius, *Crania Suecica Antiqua. Eine Darstellung der schwedischen Menschen-Schädel aus dem Steinzeitalter, dem Bronzezeitalter und dem Eisenzeitalter sowie ein Blick auf die Forschungen über die Rassencharaktere der europäischen Völker* (Aftonbladets Druckerei, 1900).

113. J. Orschiedt, A. Häußer, M. N. Haidle, K. W. Alt, C. H. Buitrago-Téllez, Survival of a multiple skull trauma: The case of an early Neolithic individual from the LBK enclosure at Herxheim (Southwest Germany). *Int. J. Osteoarchaeol*. **13**, 375–383 (2003).

114. R. J. Schulting, Claddedigaethau mewn ogofâu: Prehistoric human remains (mainly) from the caves of Wales. *Proceedings of the University of Bristol Spelaeological Society* **28**, 185–219 (2020).

115. D. Lawrence, *Orkney's First Farmers. Reconstructing Biographies from Osteological Analysis to Gain Insights into Life and Society in a Neolithic Community on the Edge of Atlantic Europe*, PhD Thesis (University of Bradford, 2012).

116. L. Fibiger, "Osteoarchaeological analysis of human skeletal remains from 23 Irish caves" in *Underground Archaeology. Studies on Human Bones and Artefacts from Ireland's Caves*, M. A. Dowd, Ed. (Oxbow Books, 2016), pp. 3–37.

117. T. Fernández-Crespo, New evidence of Early Chalcolithic interpersonal violence in the Middle Ebro Valley (Spain): two arrowhead injuries from the swallet of Las Yurdinas II. *Int. J. Osteoarchaeol*. **27**, 76–85 (2017).

118. R. J. Schulting, H. Sebire, J. E. Robb, On the road to Paradise: new insights from AMS dates and stable isotopes at Le Déhus, Guernsey, and the Channel Islands Middle Neolithic. *Oxford Journal of Archaeology* 29, 149–173 (2010).

119. S. Leach, "Odd one out? Earlier Neolithic deposition of human remains in caves and rock shelters in the Yorkshire Dales" in *Deviant Burial in the Archaeological Record*, E. Murphy, Ed. (Oxbow, 2008), pp. 35–56.

120. B. Marquez et al., "Projectile points as signs of violence in collective burials during the 4th and the 3rd millennia cal. BC in the north-east of the Iberian peninsula" in *Prehistoric Technology: 40 Years Later*, L. Longo, N. Skakun, Eds. (Archaeopress, 2005), pp. 321–325.

121. M. Lodewijckx, "Frontier settlements of the LBK in central Belgium" in *Creating Communities. New Advances in Central European Neolithic Research*, D. Hofmann, P. Bickle, Eds. (Oxbow, 2009), pp. 32–49.

122. T. Fernández-Crespo, An arrowhead injury in a Late Neolithic/Early Chalcolithic human cuneiform from the rockshelter of La Peña de Marañón (Navarre, Spain). *Int. J. Osteoarchaeol*. **26**, 1024–1033 (2016).

123. M. Wysocki, A. Whittle, Diversity, lifestyles and rites: New biological and archaeological evidence from British Earlier Neolithic mortuary assemblages. *Antiquity* **74**, 591–601 (2000).

124. C. Polet, O. Dutour, Étude paléopathologique des squelettes de l'abri des Autours (Province de Namur, Belgique). *Anthropologica et Praehistorica* **118**, 127–160 (2007).

125. C. Meyer et al., "Eine komplexe Mehrfachbestattung der Salzmünder Kultur" in *3300 BC. Mysteriöse Steinzeittote und ihre Welt*, H. Meller, Ed. (Nünnerich-Asmus, 2013), pp. 290–299.

126. J. Armendariz, S. Irigarai, F. Etxeberria, New Evidence of Prehistoric Arrow Wounds in the Iberian Peninsula. *Int. J. Osteoarchaeol*. **4**, 215–222 (1994).

127. E. Smits, "Interpersonal violence in the Late Mesolithic and Middle Neolithic in the Netherlands" in *Sticks, Stones, and Broken Bones. Neolithic Violence in a European Perspective*, R. J. Schulting, L. Fibiger, Eds. (Oxford University Press, 2012), pp. 191–206.

128. N. J. Conard, P. M. Grootes, F. H. Smith, Unexpectedly recent dates for human remains from Vogelherd. *Nature* **430**, 198–201 (2004).

129. S. Walsh, C. Knüsel, N. Melton, A re-appraisal of the Early Neolithic human remains excavated at Sumburgh, Shetland, in 1977. *Proceedings of the Society of Antiquaries of Scotland* **141**, 3–17 (2011).

130. J. Wahl, H. G. König, Anthropologisch-traumatologische Untersuchung der menschlichen Skelettreste aus dem bandkeramischen Massengrab bei Talheim, Kreis Heilbronn. *Fundberichte aus Baden-Württemberg* **12**, 65–193 (1987).

131. C. Polet, O. Dutour, R. Orban, I. Jadin, S. Louryan, A healed wound caused by a flint Arrowhead in a Neolithic human innominate from the Trou Rosette (Furfooz, Belgium). *Int. J. Osteoarchaeol*. **6**, 414–420 (1996).

132. A. Shbat, I. Růžičková, P. Herlová, Skeletal health of Late Neolithic populations from Bohemia. *Anthropologie* **47**, 195–214 (2009).

133. A. Whittle, A. Bayliss, M. Wysocki, Once in a lifetime: the date of the Waylands Smithy long barrow. *Cambridge Archaeological Journal* **17**(S1), 103–121 (2007).

134. M. J. Allen et al., Wor Barrow, *Cranborne Chase, Dorset. Chronological Modelling* (Historic England, 2016).

135. E. M. During, L. Nilsson, Mechanical surface analysis of bone: a case study of cut marks and enamel hypoplasia on a Neolithic cranium from Sweden. *Am. J. Phys. Anthropol*. **84**, 113–125 (1991).

136. M. Nunez, Cannibalism on Pitted Ware Åland? K*arhunhammas* **16**, 61–68 (1995).

.
